# Retrospective analysis of arginase 1 deficiency progression in adults over 5 years at a single metabolic centre

**DOI:** 10.1002/jmd2.12450

**Published:** 2024-09-10

**Authors:** Reena Sharma, John Bassett, Karolina M. Stepien, Andrew Oldham, Ana Jovanovic, Alison Woodall, Diane Green

**Affiliations:** ^1^ Salford Royal Foundation NHS Trust Salford UK

**Keywords:** ammonia scavengers, arginase 1 deficiency (ARG1‐D), enzyme replacement therapy, hyperargininaemia, pegzilarginase, urea cycle disorders

## Abstract

**Background:**

The clinical presentation of ARG1‐D is characterised by elevated arginine levels leading to neurological and mobility impairments. Information about long‐term outcomes in adults is lacking, which prompted us to undertake a retrospective observational study.

**Methods:**

We extracted ARG1‐D patient data spanning a 5‐year period from electronic health records. Ethical approval was not required for the study. Informed consent was obtained.

**Results:**

We identified nine ARG1‐D patients from consanguineous backgrounds. Age of symptom onset ranged from infancy to age 7 years, age of diagnosis from infancy to 20 years. Patients had paraparesis or altered gait of varying degree and had experienced early ARG1‐D onset. Over 5 years, mobility declined in six (6/9, 67%) patients. Three patients (3/9, 33%) were fully dependent and hoisted. Two (2/9, 22%) reached adulthood before experiencing hyperammonaemia, another one (1/9, 11%) first experienced hyperammonaemia at age 15 years. One patient (1/9, 11%) started on ammonia scavenger therapy in adulthood, one (1/9, 11%) required a second scavenger to be added to their treatment regimen. Two patients (2/9, 22%) had gastrostomy tubes inserted for nutrition and supplements at age 9 years and 15 years. Six patients (6/9, 67%) had raised levels of ALT; of these, four (4/9, 44%) also had elevated AFP. Heterogeneity of ARG1‐D symptoms was evident, suggesting complex genetic and environmental interactions.

**Conclusion:**

ARG1‐D presents significant lifelong challenges with deteriorating mobility and more frequent metabolic crises. Current management strategies are insufficient for preventing progression, highlighting the need for innovative treatments like enzyme replacement and gene therapy.

**Synopsis**

This paper describes the long‐term progression of ARG1‐D in adults, despite the use of best standard of care, highlighting the urgent need for new treatment options.

## 1 INTRODUCTION

Arginase 1 deficiency (ARG1‐D; OMIM 207800) is a rare progressive inborn error of metabolism of the urea cycle caused by mutations in the *ARG1* gene leading to impaired or absent arginase 1 activity.^1^ This is the final enzyme in the urea cycle pathway that converts arginine to ornithine and urea.^2^ Lack of arginase activity results in arginine and its metabolites accumulating in the liver initially and then in other organs including the central nervous system and cerebrospinal fluid.^3^

In the general population, plasma arginine levels range from 20 to 100 μmol/L, while in people with ARG1‐D they are two‐ to six‐fold higher than normal. The literature reports levels typically >300 μmol/L, although levels >1000 μmol/L have been reported.^4^ Hyperargininaemia typically manifests as cognitive, neurological and mobility issues^5^ including spastic paraparesis in 80%–90% of patients.^6^ Progressive neurological and motor deterioration affects mobility and delays growth and cognitive development.^2^ Seizures occur in 60%–75% of patients.^6^, ^7^ The clinical presentation and rate of progression of the condition varies^5^ even with the use of low protein diets and ammonia scavengers. The phenotype seems to be determined by the homozygosity or compound heterozygosity of previously identified loss of function or missense variants.^8^

ARG1‐D is the least common of all UCDs, with estimated incidence of approximately 1 in 726 000.^9^ While hyperammonaemia is a common feature of the other UCDs associated with enzyme deficiencies earlier in the pathway,^5^, ^10^, ^11^, ^12^ it is underappreciated in ARG1‐D. Current standard or care (SOC) includes protein restriction to achieve plasma arginine level less than 200 μmol/L with ammonia scavenger therapies. These are unable to resolve all symptoms, particularly neurological progression, with not much distinction in patients diagnosed earlier or later in life.^3^, ^4^ Better outcomes can be achieved with liver transplantation which when undertaken in patients at a young age can prevent neurological damage and improve quality of life.^13^ Enzyme replacement therapy has also shown to provide benefit, through normalisation of plasma arginine and an improvement in functional mobility.^14^ Pre‐clinical studies investigating the potential of an ARG1 mRNA therapy to treat ARG1‐D found an increase in functional protein expression of ARG1 and an increase in urea, suggesting ARG1 mRNA therapy could be a potential future treatment option for ARG1‐D.^15^, ^16^

Although the clinical presentation of ARG1‐D has been reasonably well documented, there is a paucity of information about long‐term outcomes in adults. To help address this, we present data describing symptom progression and risks for acute illness in adult ARG1‐D patients at our specialist metabolic centre.

## 2 METHODS

### 2.1 Study design

This was a retrospective observational study in adult ARG1‐D patients at our inherited metabolic disease centre in the UK (Salford Royal Foundation NHS Trust) from 2017 onwards with a follow up of 2 to >5 years at our centre. Information was extracted from electronic health records. Ethical approval was not required. Written consent was sought from all patients or their legal representatives.

### 2.2 Study variables

#### 2.2.1 Participants

Baseline data included age, sex, weight, Body Mass Index, ethnicity, the presence of pathogenic variants, and clinical history. Information regarding initial symptoms, learning disabilities, schooling, speech difficulties, epilepsy, mode of diagnosis, and need for carers was collected.

#### 2.2.2 Mobility and treatments

Data regarding physical capabilities included the ability to move independently, gait quality, use of walking aids, and wheelchair reliance. Medical interventions, bone health, and occurrence of bone fractures were recorded. Changes in mobility were assessed at appointments with physiotherapists.

#### 2.2.3 Nutritional assessment

Dietary intake was assessed by calculating the daily protein intake. We evaluated protein tolerance and use of supplements and ammonia scavengers. Hospital admissions and emergency department visits were documented.

#### 2.2.4 Laboratory measurements

Blood samples were analysed for levels of ammonia, arginine, ornithine, glutamine, glycine, alpha‐fetoprotein (AFP) and alanine transaminase (ALT).

### 2.3 Statistical analysis

Generation of descriptive statistics was undertaken using Microsoft Excel.

## 3 RESULTS

Nine adult patients with ARG1‐D were identified from five consanguineous families of different ethnicities (Table 1). Age of symptom onset ranged from infancy up to age 7 years and the age of diagnosis from infancy up to 20 years. One patient died of pulmonary embolism, which may have been indirectly related to arginase 1 deficiency.

**TABLE 1:** Patient characteristics.

	Pt1	Pt2	Pt3	Pt4	Pt5	Pt6	Pt7	Pt8	Pt9
Age at end of follow up (years)	34	20+	29	31	27	22	27	24	18 (16 at start of follow‐up)
Sex	M	F	F	F	M	F	F	F	F
Weight (kg) (BMI—kg/m^2^) at end of follow up	103.6 (36.7)	107.8 (44.9)	76 (22.6)	52 (not known)	22.8 (not known)	34 (not known)	88 (38.2)	56 (not known)	67.8 (27.9)
Ethnicity	Pakistan	Pakistan	Pakistan	Yemen	Yemen	Yemen	Pakistan	Pakistan	Somalia
Mode of diagnosis	Enzymatic analysis (erythrocyte arg 1 activity)	Enzymatic analysis (erythrocyte arg 1 activity)	Enzymatic analysis (erythrocyte arg 1 activity)	Genetic (next generation sequencing)	Genetic (next generation sequencing)	Genetic (next generation sequencing)	Enzymatic analysis (erythrocyte arg 1 activity)	Enzymatic analysis (erythrocyte arg 1 activity)	Enzymatic analysis (erythrocyte arg 1 activity)
Gene variant (via *ARG1* gene testing)	Not known (family have not consented to testing)	Not known (family have not consented to testing)	Not known (family have not consented to testing)	c.370G>T (p.Asp124Tyr)	c.370G>T (p.Asp124Tyr)	c.370G>T (p.Asp124Tyr)	c.93del (p.Arg32Glufs*16)	c.802+2T>G	c.23T>A (p.Ile8Lys)
Age symptom onset (years)	3	7	3	6	3	3	6	Neonate	1
Age at diagnosis (years)	14	11	9	25	21	16	7	3 months	2
Symptom at onset	Delayed speech	Mild learning difficulty	Epilepsy	Abnormal gait	Abnormal gait	Abnormal gait	Episodic behavioural changes	Pneumothorax and unwell	Global developmental delay
Learning disability^a^	Yes (very mild)	Yes	Yes	Yes	Yes	Yes	Yes (very mild)	Yes	Yes
Mental health input (including whether on anti‐depressants/anti‐psychotics)	No	No	No	No	No	No	Seen by neuropsycology	No	No
Special needs schooling	Extra support main school	Extra support main school	Yes	Yes	Yes	Yes	Extra support main school	Yes	Yes
Able to consent to study without legal representative?	No	No	No	No	No	No	Yes	No	No
Speech difficulties	No	No	Yes	Yes	Yes	Yes	No	Yes	Yes
Epilepsy	Yes	No	Yes	No	No	No	No	Yes	No
Level of dependence (modified ranking scale)	No significant disability	No significant disability (at time of death)	Severe disability.	Moderate severe disability	Moderate severe disability	Moderate severe disability	Moderate disability	Severe disability	Moderate disability
Admissions in last 5 years (all causes)	0	N/A	0	3	1	1	7	1	3
Reported use of emergency regimen in last 5 years	1	N/A	0	10	2	2	8	10	5

Abbreviation: N/A, not available.

^a^ Based on caring/educational requirements rather than neurocognitive testing.

All patients had paraparesis or altered gait of varying degree and had experienced early onset of ARG1‐D (Table 2). Follow up of over a five‐year period found declining mobility in six (6/9, 67%) patients. The other three patients (3/9, 33%) were fully dependent and hoisted at the start of this period and remained so over the next 5 years.

**TABLE 2:** Physical characterisation, mobility, and interventions.

	Pt 1	Pt 2	Pt 3	Pt 4	Pt 5	Pt 6	Pt 7	Pt 8	Pt 9
Mobile	Yes	Yes	No	No	No	No	Yes	No	Yes
Gait	Normal	Normal	N/A	N/A	N/A	N/A	Scissor gait	N/A	Wide‐based
Walking aid	No	No	N/A	N/A	N/A	N/A	Zimmer frame	N/A	No
Wheelchair use	No	No	Yes	Yes	Yes	Yes	Yes	Yes	No
Transfer hoist	Yes	Yes	No	Yes	No	Yes	Yes	No	Yes
Oral baclofen	Yes	Yes	Yes	Yes	Yes	Yes	Yes	No	No
Baclofen pump	No	No	No	Yes	No	Yes	No	No	No
Botulinum toxin	No	No	No	Yes	Yes	Yes	No	No	No
Tendon surgeries	No	No	No	No	Yes	Yes	Y	No	No
Bone fractures	No	No	No	No	No	No	Y	No	No
*Z* score lumbar spine	1.6	N/A	−1.6	−3.8	−6.6	−4.7	−2.5	−1.6	N/A
*Z* score total hip	1.5	N/A	−0.8	−3.9	N/A	−4.3	N/A (hip dysplasia)	−2.1	N/A
*Z* score neck of femur	1.5	N/A	−2.1	−3.0	N/A	−3.8	N/A (hip dysplasia)	N/A	N/A
*Z* score forearm	N/A	N/A	N/A	N/A	−5.9	N/A	N/A	−0.2	N/A
Worsening mobility over last 5 years	Stable	N/A	Stable	Declining	Declining	Declining	Declining	Declining	Declining

Abbreviation: N/A, not available.

One patient started on ammonia scavenger therapy in adulthood and one patient required a second ammonia scavenger to be added to their treatment regimen (Table 3). Two patients reached adulthood before experiencing their first episodes of hyperammonaemia and another patient experienced their first episode of hyperammonaemia at age 15 years. Two patients (2/9, 22%) had gastrostomy tubes inserted for nutrition and medication supplements at age 9 years and 15 years. Six (6/9, 67%) had raised levels of ALT, and of these, four (4/9, 44%) also had elevated AFP. Of the five patients who underwent a liver scan, one (1/5, 20%) showed cirrhosis, three (3/5, 60%) had a fatty liver plus one had a gall stone, and one (1/5, 20%) had multiple non‐malignant nodules. Ornithine in the group was low or towards the lower end of normal. Compliance with dietary management and ammonia scavengers was generally good but was better in the three patients diagnosed before age of 7 than those diagnosed later in life. Decompensations in this cohort were mostly due to illness.

**TABLE 3:** Medical management and laboratory data.

	Pt 1	Pt 2	Pt 3	Pt 4	Pt 5	Pt 6	Pt 7	Pt 8	Pt 9
Diet									
Recent protein intake (g/kg)	0.33–0.6	0.23	0.40–0.52	0.69	0.73	1.24	0.57	0.58	0.82
Worsening protein tolerance over 5 years (g/kg)	N	N	N	N	Y (1.23–0.73) 40% reduction	N	N	Y (0.61–0.58) 5% reduction	N
Diet supplements	Nil	Nil	Nil	Dialamine	Dialamine Calogen	Dialamine	Basecal EAA	Paedisure	Dialamine Nutrison
Ammonia scavenger	Nil	Nil	Nil	GPB	None	None	NaPheBut NaBenz Carnitine	GPB NaBenz	GPB NaBenz Carnitine
Laboratory data									
Ammonia (reference range 12–32 μmol/L)	Mean: 78 Range: 26–108 SD: 34	N/A	Mean: 62 Range: 29–92 SD: 32	Mean: 79 Range: 18–145 SD: 54	Mean: 29 Range: 21–42 SD: 9	Mean: 26 Range: 17–42 SD: 9	Mean: 118 Range: 24–161 SD: 74	Mean: 141 Range: 40–197 SD: 44	Mean: 200 Range: 45–294 SD: 94
Arginine (reference range 22–100 μmol/L), 2019–2023	Mean: 746 Range: 787–819 SD: 82	Mean: 656 Range: 642–673 SD:13	Mean: 835 Range: 748–922 SD: 87	Mean: 156 Range: <20 517 SD: 196 On trial mean: 41, SD: 68	Mean: 556 Range: 525–684 SD: 84	Mean: 577 Range: 413–638 SD: 99	Mean: 384 Range: 230–506 SD: 86	Mean: 600 Range: 578–621 SD: 22	Mean: unknown, results from another hospital Range: 463–633
Ornithine (reference range 40–150 μmol/L), 2019–2023	Mean: 44 Range: 49–38 SD: 6	Mean: 28 Range: 25–29 SD: 3	Mean: 40.5 Range: 29–52 SD: 16	Mean: 75.6 Range: 19–190 SD: 80	Mean: 30 Range: 29–39 SD: 7	Mean: 27 Range: 2–27 SD: 2	Mean: 18 Range: 11–22 SD: 6	Mean: 16 Range: 10–21 SD: 8	Mean: N/A Range: N/A–33 SD: N/A
Glutamine (reference range 390–740 μmol/L), 2019–2023	Mean: 516 Range: 524–607 SD: 95	Mean: 484 Range: 466–501 SD: 18	Mean: 554 Range: 495–613 SD: 83	Mean: 502 Range: 420–652 SD: 88	Mean: 508 Range: 390–691 SD: 132	Mean: 633 Range: 512–751 SD: 120	Mean: 242 Range: 329–297 SD: 110	Mean: 583 Range: 532–633 SD: 71	Mean: N/A Range: N/A–353 SD: N/A
Glycine (reference range 160–400 μmol/L), 2019–2023	Mean: 99 Range: 38–144 SD: 55	Mean: 184 Range: 155–231 SD: 41	Mean: 189 Range: 144–234 SD: 64	Mean: 178 Range: 134–293 SD: 68	Mean: 204 Range: 133–333 SD: 90	Mean: 179 Range: 154–217 SD: 33	Mean: 100 Range: 72–127 SD: 28	Mean: 178 Range: 120–235 SD: 81	Mean: N/A Range: N/A–108 SD: N/A
AFP (reference range < 6.7 kU/L), 2019–2023	Mean: N/A Range: N/A–3.3 SD: N/A	N/A	Mean: N/A Range N/A–<1.1 SD: N/A	Mean: 23.5 Range: 14–26 SD: 6	Mean: 4.7 Range: 3.9–5.5 SD: 1	Mean: 9.4 Range: 7.8–11 SD: 2	Mean: 54 Range: 52–56 SD: 2	Mean: N/A Range N/A–<1.1 SD: N/A	Mean: N/A Range; N/A–33 SD: N/A
ALT (reference range 7–40 U/L), 2019–2023	Mean: 30 Range: 29–34 SD: 4	Mean: 28 Range: 24–32 SD: 2	Mean: 23 Range: 23–23	Mean: 68 Range: 46–76 SD: 14	Mean: 48 Range: 40–59 SD: 9	Mean: 54 Range: 42–64 SD: 9	Mean: 51 Range: 47–54 SD: 4	Mean: 80 40–104 SD: 28	Mean: N/A Range: N/A–219 SD: N/A

Abbreviations: EAA, essential amino acids; GPB, glycerol phenylbutyrate (Ravicti®, Immedica Pharma AB); N/A, not available; NaBenz, sodium benzoate; NaPheBut, sodium phenylbutyrate comprising unbranded formulations or Ammonaps® (Immedica Pharma AB).

Due to the significant heterogeneity of the disease, further information for each individual family is provided below.

### 3.1 Family 1 (Patients 1–3)

All siblings had learning difficulties and required special schooling or extra support. They were diagnosed by RBC arginase 1 activity and raised arginine levels when the eldest was age 13 years. Two siblings (Patients 1 and 3) had epilepsy. Mobility was most affected in the youngest sibling. Patient 2 died from a pulmonary embolism following a long flight during the follow up period. This may have been indirectly related to ARG1‐D due to venous stasis resulting from his poor mobility.

### 3.2 Family 2 (Patients 4–6)

Two siblings were noticed to have delay and difficulty in walking. The youngest had normal milestones until age 3 years. All experienced difficulties with speech, had learning difficulties, and went to a special needs school. All were using wheelchairs by 8 years. Diagnosis was by whole genome sequencing when the eldest was age 20 years. The siblings have undergone tendon‐release surgeries and botulinum toxin and baclofen pump to help with muscle spasticity. Only Patient 4 was able to walk more than a metre 5 years ago; the patient participated in a clinical trial of an enzyme replacement therapy (pegzilarginase) and remained stable during the 2‐year trial period with arginine levels of ~50 μmol/L. Since the discontinuation of therapy, the patient has become fully wheelchair dependent and has new onset dystonia with a rise in arginine to >500 μmol/L. Two of the siblings are on a soft diet due to swallowing difficulties. Impaired growth and pubertal development were identified in the male sibling, Patient 5, related to hypogonadotropic hypogonadism. The patient required testosterone therapy from age 16 years for 10 years. The patient also has cirrhosis of the liver and is undergoing further investigations.

### 3.3 Family 3 (Patient 7)

Patient 7 was diagnosed when presenting with hyperammonaemia leading to periods of behaviour changes and change in gait at age 7 years. The family had a novel mutation for the condition.^16^ At age 10 years, the patient was found to have renal tubulopathy and since then has been receiving sodium bicarbonate, phosphate, and potassium supplements. At age 25 years, the patient was noticed to have pancytopenia of a consumptive nature with no cause identified, and so now receives recombinant granulocyte colony‐stimulating factor (filgrastim) to maintain the blood count. At age 26, the patient sustained multiple fractures of both legs following a low impact fall that required surgical fixation and plaster cast with immobilisation. A DXA scan showed significantly reduced bone density. The patient is mobile with crutches indoors and uses a wheelchair outdoors. A magnetic imaging resonance (MRI) scan of the liver showed multiple non‐malignant lesions with raised alpha fetoprotein (AFP) and C‐reactive protein (CRP). The patient is currently being considered for liver transplant.

The patient's two younger siblings were diagnosed at birth via family cascade screening but were never seen in the adult service. The youngest had normal mental capabilities and normal gait but a severe electrolyte imbalance due to renal tubulopathy requiring IV repletion. The patient died of septicaemia at the age of 5 years. The second patient had near normal neurodevelopment and gait but developed intractable high ammonia and encephalopathy at the age of 15 years which did not respond to conservative management. The patient required an urgent liver transplant but died post‐operatively because of fungal septicaemia.

### 3.4 Family 4 (Patient 8)

Patient 8 was the only affected member in the family. The patient presented with a pneumothorax and respiratory distress in the newborn period. At age 1 year, during routine follow up, development delay was noticed, and investigations led to the diagnosis of ARG1‐D. The patient was commenced on protein restriction and ammonia scavenger therapy. At age 2 years she developed seizures. Gastrostomy was inserted at age 9 years because of poor feeding. The patient was very prone to vomiting. Hyperammonaemia was managed mainly at home with an oral emergency regimen. Her seizures worsened over the last 2 years.

### 3.5 Family 5 (Patient 9)

This index patient was investigated for global developmental delay and was diagnosed at age 2 years. Despite dietary restriction, the patient's arginine levels remained above 200 μmol/L. From pubertal age, the patient started experiencing episodes of hyperammonaemia. At age 15 years, the patient required ITU admission with hyperammonaemia encephalopathy. Gastrostomy was inserted for emergency management. The patient continues to have episodes of metabolic decompensation mostly managed at home. The patient has a wide‐based gait with hypertonia of plantar flexion. They have young siblings with the same condition.

## 4 DISCUSSION

ARG1‐D is a debilitating and progressive disease in adults. Lower limb spasticity and a changed gait was a universal feature in our cohort, and there was a continued decline in mobility into adulthood. None of our patients were able to sustain levels of arginine below the 200 μmol/L target. This finding, coupled with the clinical data, demonstrated that current SOC is inadequate. Contribution from endogenous production of arginine also makes it challenging to achieve the target levels.

Approximately half of our patients were having episodes of metabolic decompensation requiring hospital admissions or using their emergency regimen at home, with hyperammonaemia being a significant, underappreciated risk in ARG1‐D. Hyperammonaemia became more prevalent when patients reached adulthood. Two patients had hyperammonaemia for the first time in adulthood and another patient in adolescence. One patient required ammonia scavenger therapy for the first time in adulthood and one required addition of a second ammonia scavenger agent.

Only one of the patients from our cohort died during the follow‐up period at age 20 years, from a pulmonary embolism, which may have been precipitated by venous stasis from reduce mobility secondary to ARG1‐D. However, it is to be noted that two paediatric siblings from Family 3—not included in the adult data—died due to causes related to ARG1‐D; one from intractable hyperammonaemia encephalopathy (ammonia level approximately 600 μmol/L, unresponsive to conservative management) following urgent liver transplant at age 15 years, and another at age 5 years requiring daily IV electrolytes for tubulopathy, due to septicaemia. This represents a mortality rate of more than 25% for this cohort (if the younger siblings of Family 3 are included).

Information on bone density has not been reported before. DXA results for seven patients showed one patient with normal bone density, four with osteoporosis and two with osteopenia. DXA for the hip was not always successful due to hip dysplasia, positioning, and artefacts. Caution is required in the interpretation of DXA results for these patients. To accommodate the difficulties involved with hip scans, bone density at radius could be regularly considered instead along with spine. All patients were replete with vitamin D.

We identified five patients with liver abnormalities on imaging and raised AFP. This is consistent with the findings of chronic hepatocellular injury observed in both ARG1‐D and arginosuccinate lyase deficiency (ASLD).^17^ Raised AFP and hepatocellular carcinoma has also been reported in ARG1‐D.^18^ Therefore hepatic abnormalities and raised AFP require careful monitoring in adults, and hepatology review should be requested where appropriate.

Females reached menarche at the appropriate age. One of the two male patients was identified with hypogonadotropic hypogonadism requiring replacement therapy. No other endocrine issues were noted; this aspect has not been reviewed in the past.

Pancytopenia was seen in one patient with regular need of filgrastim. It could be hypothesised that there is expression of arginase 1 in myeloid cells which could be responsible for this,^19^ or it could be due to consumption due to multiple hepatic adenomas. The patient's two siblings diagnosed at birth had a much more severe and tragic clinical course. The family had a novel mutation for the *ARG1* gene.^20^ Two members from the same family also had renal tubulopathy requiring regular IV or oral replacement therapy which has not been described in ARG1‐D. This was investigated by the paediatric team but no clear cause was found. It may be tubulopathy represents another co‐morbid condition given the family's consanguinity.

Swallowing difficulties and poor oral intake could lead to insertion of gastrostomy and modification of the diet. Two patients were on modified diets due to swallowing difficulties; one required gastrostomy at age 9 years because of poor feeding, another one required gastrostomy at age 15 years for medications and home emergency regimen. Recurrent episodes of vomiting have been noticed in Families 3–5 and have been the cause of visits to the emergency department or admission to hospital.

Guanidino compounds have been proposed as a cause of neurological disease in this condition.^21^ Ornithine levels were low or towards the lower end of normal in our cohort. Further work could explore whether ornithine supplements would lead to a change in the disease course. The one patient who had been part of the clinical trial of enzyme replacement therapy had worsened neurologically prior to the trial, stabilised during the trial and deteriorated with new onset dystonia a few months after the trial ended.

In summary there are ongoing challenges with this cohort of patients as adults. The hepatic, gastrointestinal, and bony complications appear later in the course of the disease and need to be monitored. Protein tolerance declines for some in adulthood and adults are more prone to catabolism—accordingly, patients are more likely to present with episodes of high ammonia and metabolic decompensation. The current standard of care is not effective. An enzyme replacement therapy, pegzilarginase (Loargys®, Immedica Pharma AB), has recently been approved in the EU and Great Britain for the treatment of arginase 1 deficiency in patients aged 2 years and older. It may provide a means of altering the disease course for these patients. Real‐world studies are required.

## INFORMED CONSENT STATEMENT

Written consent was obtained from all patients.

## AUTHOR CONTRIBUTIONS

Reena Sharma conceived the work, Andrew Oldham, Karolina M Stepien, Diane Green, Alison Woodall, and Reena Sharma collected the data, undertook data analysis and interpretation, and drafted the article, and Reena Sharma, John Bassett, Diane Green, Andrew Oldham, Karolina M Stepien, and Ana Jovanovic critically reviewed the article. Karolina M Stepien also drafted the introduction. All authors reviewed the final manuscript.

## CONFLICT OF INTEREST STATEMENT

Reena Sharma was a principal investigator for a clinical study funded by Aeglea Bio Therapeutics investigating the efficacy and safety of pegzilarginase in arginase 1 deficiency. John Bassett, Karolina Stepien, Andrew Oldham, Ana Jovanovic, Alison Woodall, and Diane Green declare no conflicts of interest.
